# Comparison of regional skeletal muscle tissue oxygenation in college athletes and sedentary control subjects using quantitative BOLD MR imaging

**DOI:** 10.14814/phy2.12903

**Published:** 2016-08-17

**Authors:** Mitchel R. Stacy, Christopher M. Caracciolo, Maolin Qiu, Prasanta Pal, Tyler Varga, Robert Todd Constable, Albert J. Sinusas

**Affiliations:** ^1^ Department of Internal MedicineYale University School of MedicineNew HavenConnecticut; ^2^ Department of Radiology and Biomedical ImagingYale University School of MedicineNew HavenConnecticut; ^3^ Department of NeurosurgeryYale University School of MedicineNew HavenConnecticut

**Keywords:** Blood oxygen level dependent, exercise training, magnetic resonance imaging, tissue oxygenation, vascular function

## Abstract

Blood oxygen level‐dependent (BOLD) magnetic resonance (MR) imaging permits noninvasive assessment of tissue oxygenation. We hypothesized that BOLD imaging would allow for regional evaluation of differences in skeletal muscle oxygenation between athletes and sedentary control subjects, and dynamic BOLD responses to ischemia (i.e., proximal cuff occlusion) and reactive hyperemia (i.e., rapid cuff deflation) would relate to lower extremity function, as assessed by jumping ability. College football athletes (linemen, defensive backs/wide receivers) were compared to sedentary healthy controls. BOLD signal of the gastrocnemius, soleus, anterior tibialis, and peroneus longus was assessed for peak hyperemic value (PHV), time to peak (TTP), minimum ischemic value (MIV), and time to recovery (TTR). Significantly higher PHVs were identified in athletes versus controls for the gastrocnemius (linemen, 15.8 ± 9.1%; defensive backs/wide receivers, 17.9 ± 5.1%; controls, 7.4 ± 3.5%), soleus (linemen, 25.9 ± 11.5%; backs/receivers, 22.0 ± 9.4%; controls, 12.9 ± 5.8%), and anterior tibialis (linemen, 12.8 ± 5.3%; backs/receivers, 12.6 ± 3.9%; controls, 7.7 ± 4.0%), whereas no differences in PHV were found for the peroneus longus (linemen, 14.1 ± 6.9%; backs/receivers, 11.7 ± 4.6%; controls, 9.0 ± 4.9%). In all subject groups, the gastrocnemius and soleus muscles exhibited the lowest MIVs during cuff occlusion. No differences in TTR were found between muscles for any subject group. PHV of the gastrocnemius muscle was significantly and positively related to maximal vertical (*r *= 0.56, *P* = 0.002) and broad jump (*r *= 0.47, *P* = 0.01). These results suggest that BOLD MR imaging is a useful noninvasive tool for evaluating differences in tissue oxygenation of specific muscles between active and sedentary individuals, and peak BOLD responses may relate to functional capacity.

## Introduction

The beneficial effects of exercise training on cardiovascular health have been well documented, and evidence suggests that many of the health‐related benefits associated with exercise are modulated by repetitive increases in vessel wall shear stress, which may result in improved vascular function as well as positive vascular remodeling (Green et al. 2012). Studies evaluating vascular adaptation in response to chronic aerobic exercise have demonstrated that exercise training leads to an increased size of peripheral conduit arteries (Huonker et al. 2003; Schmidt‐Trucksass et al. 2000; Zeppilli et al. 1995), increased microvascular density of skeletal muscle (Cocks et al. 2013), and improved vasodilator capacity of both peripheral (Martin et al. 1987; Sinoway et al. 1986; Snell et al. 1987) and coronary vessels (Hildick‐Smith et al. 2000; Pelliccia et al. 1990).

Skeletal muscle is composed of several fiber types, which all possess different contractile and metabolic properties and can be generally classified as slow twitch (ST) and fast twitch (FT) (Engel 1962). In humans, FT fibers are further subdivided into FT_a_ fibers, which are more aerobic (or oxidative), and FT_b_ fibers, which are more anaerobic (or glycolytic) fibers (Brooke and Kasier 1970). A large amount of heterogeneity in muscle fiber type composition can exist between people due to the ability of skeletal muscle tissue to adapt to a multitude of variables, such as exercise training, which can lead to changes in muscle mass, strength, and composition (i.e., increased vascular density), and potentially explain some of the differences in fiber types that exist between athletes who undergo different training programs (Costill et al. 1976; Fink et al. 1977; Saltin et al. 1977). Noninvasive imaging is commonly applied to evaluate the effects of exercise training on skeletal muscle and the peripheral vasculature, with the majority of research directed at the application of ultrasound to assess skeletal muscle blood flow and vascular reactivity (Green et al. 2012). Blood oxygen level‐dependent (BOLD) magnetic resonance (MR) imaging is a noninvasive technique that can be used to evaluate dynamic changes in skeletal muscle oxygenation in response to various stimuli, such as reactive hyperemia (Ledermann et al. 2006a; Schulte et al. 2008; Utz et al. 2005) or muscle contraction (Towse et al. 2011, 2005). BOLD MR image signal intensity is based on relative changes in paramagnetic deoxyhemoglobin and diamagnetic oxyhemoglobin in the microcirculation of skeletal muscle, where an increase in blood oxygen saturation results in an increase in T2*‐weighted BOLD image signal intensity (Ledermann et al. 2006b). Due to the sensitivity of BOLD for detecting dynamic changes in intravascular hemoglobin oxygenation, the BOLD image signal intensity can also be affected by multiple factors, such as blood volume, fluid shifts, metabolic factors, vascular architecture, and magnetic field angulation; however, fluctuations in BOLD signal intensity are believed to be largely dependent on dynamic changes in blood flow within the microcirculation (Jacobi et al. 2012). Changes in BOLD image signal intensity have been shown to correlate well with laser Doppler flow and transcutaneous oxygen pressure (TcPO_2_) during the assessment of transient ischemia and reactive hyperemia paradigms in the lower extremities (Ledermann et al. 2006b; Partovi et al. 2013, 2014), and is in close agreement with near infrared spectroscopy (NIRS) measures of blood volume and saturation following maximal muscle contractions (Towse et al. 2011). Prior studies evaluating dynamic changes in BOLD signal intensity during reactive hyperemia have demonstrated impaired BOLD responses with increasing age (Kos et al. 2009; Schulte et al. 2008), in the setting of peripheral arterial disease (PAD) (Ledermann et al. 2006a), and in young smokers (Nishii et al. 2015).

The coregistration of functional BOLD images with anatomical MR images allows for evaluation of volumetric changes in skeletal muscle oxygenation within lower extremity muscle groups (Ledermann et al. 2006a) or vascular territories of interest (Stacy et al. 2016). Specifically, functional BOLD images have been paired with anatomical images to demonstrate impaired BOLD signal intensity responses to reactive hyperemia in PAD patients within specific calf muscles (Ledermann et al. 2006a), and have also shown significantly higher and more prolonged BOLD responses to muscle contraction in the anterior tibialis muscle of physically active individuals when compared to sedentary counterparts (Towse et al. 2005). However, BOLD MR imaging has not been applied to evaluate which lower extremity muscles undergo, or are most susceptible to, changes in tissue oxygenation following chronic exercise training. Prior work in young athletes suggests that exercise training may not improve vascular function measures above those of sedentary controls due the preexistence of healthy vessels in young individuals; however, prior work in this field has primarily focused on evaluating macrovascular, and not microvascular function (Montero et al. 2014). We hypothesized that BOLD MR imaging during transient ischemia and reactive hyperemia would allow for quantitative assessment of regional volumetric differences in lower extremity skeletal muscle oxygenation at the microvascular level between sedentary healthy control subjects and college football athletes. We also hypothesized that peak hyperemic BOLD responses would relate to lower extremity functional capacity, as assessed by maximal vertical and broad jumping ability.

## Materials and Methods

### Research subjects

Young healthy men (*n* = 33) were recruited to participate in the MR research protocol. Of the subjects recruited, 23 were college football athletes of two distinctly different playing positions, which consisted of linemen (*n* = 12) and defensive backs/wide receivers (*n =* 11). Linemen, whose main objective in American football is to block and/or push opposing linemen off the line of scrimmage, are characterized by larger body sizes and high levels of upper and lower body strength, which is typically utilized during quick bursts. By contrast, the role of backs and receivers is to advance the football by running with the ball or passing it, or cover opposing players. Therefore, these athletes are characterized by smaller body sizes and are generally much faster to allow for covering or dodging of opposing players. In addition to football athletes, 10 sedentary control subjects were recruited for evaluating differences in skeletal muscle oxygenation between exercise‐trained and ‐untrained individuals, as well as assessment of test–retest repeatability of our BOLD imaging approach. In all subjects, maximal vertical jump was measured using a vertec vertical jump tester, and maximal broad jump was evaluated via the standing long jump test. Body fat composition was also evaluated using a three‐site skin‐fold assessment (abdomen, suprailiac, and triceps), with all measurements taken by an experienced individual. A standard medical history questionnaire was administered to evaluate each subject's risk factors for cardiovascular, pulmonary, and metabolic disease, which included history of smoking, hypertension, diabetes, hypercholesterolemia, and family history of coronary artery disease. Additionally, the International Physical Activity Questionnaire (IPAQ) was administered to evaluate each subject's activity levels for the week prior to study involvement. The study protocol was approved by the Institutional Review Board for Human Subjects Research and Review Committee, and was in accordance with the guidelines set forth by the Declaration of Helsinki. Subjects reported for imaging following an 8‐h fast that also consisted of abstinence from caffeine and alcohol. All individuals provided written informed consent after receiving an explanation of the experimental procedures and potential risks associated with participating in this study.

### Exercise training program

College football athletes recruited for the research protocol were actively involved in a structured exercise training program, which consisted of a resistance training and plyometric regimen 3 days per week, in addition to full on‐field practice sessions 5–7 days per week. Specifically, resistance training exercises that involved the lower extremities consisted of compound, dynamic full‐body barbell lifts that involved multiple joints, including the hips, knees, and ankles. Lifts consisted of the back squat (once/week, 5 sets of 2–5 repetitions), hang clean (twice/week, 3 sets of 1–2 repetitions), and clean pulls (twice/week, 5 sets of 2 repetitions). All lifts activated a large range of lower extremity muscles, including but not limited to the quadriceps, glutes, adductors, hamstrings, soleus, and gastrocnemius muscles. In addition to barbell lifts, athletes performed hamstring curls (once/week, 5 sets of 10 repetitions). Plyometric exercises consisted of two sets each of bounding exercise and the triple broad jump.

### MR imaging protocol

Sedentary control subjects reported for MR imaging on two separate occasions for repeatability testing of our quantitative BOLD analyses, with each visit being separated by at least 1 week. All athletes reported for a single visit. Resting blood pressure and heart rate measurements were manually acquired prior to the start of each imaging session. All subjects underwent BOLD MR imaging of the dominant leg at the level of the mid‐calf on a 3 Tesla MR System (TIM Trio; Siemens Healthcare, Erlangen, Germany) using spine and body phased array coils (Siemens Healthcare, Erlangen, Germany). The leg was firmly secured to restrict movement. A blood pressure cuff was secured around the thigh for rapid inflation (ischemia) and deflation (reactive hyperemia) using a Hokanson E20 AG101 Rapid Cuff Inflation System (D.E. Hokanson, Inc; Bellevue, WA). Serial BOLD images were acquired over 15 min, with the first 5 min representing baseline assessment, followed by a 5 min occlusion phase (cuff pressure inflated to 50 mmHg above resting systolic pressure), and 5 min of reactive hyperemia following cuff deflation. BOLD imaging parameters were as follows: 10 mm effective slice thickness (i.e., six slices with a slice thickness of 5 mm and a distance factor of 100%), FOV = 360 × 180 mm^2^, in‐plane matrix size = 128 × 64, TR=3000 ms, TE = 40 ms, and flip angle = 90°. Following each BOLD acquisition, coregistered high‐resolution anatomical images were acquired with a T1‐FLASH (i.e., fast low‐angle shot) gradient echo sequence to guide segmentation of calf muscles of interest. T1‐FLASH imaging parameters were as follows: 1.4 mm slice thickness, FOV = 320 × 320 mm^2^, in‐plane matrix size = 256 × 256, TR = 9.83 ms, TE = 4.9 ms, and flip angle = 10°.

### Image analysis

Volumes of interest (VOIs) were generated from BOLD MR images of the calf with the guidance of coregistered T1‐FLASH anatomical images, which assisted with identification and manual segmentation of calf muscles. This image segmentation approach has been previously applied in our laboratory for analysis of BOLD responses in the foot (Stacy et al. 2016), as well as lower extremity single photon emission computed tomography/computed tomography (SPECT/CT) perfusion imaging (Stacy et al. 2014). All image segmentation and analysis was performed using BioImage Suite (http://www.bioimagesuite.org), an image analysis toolkit.

Time‐course data were generated to evaluate dynamic changes in BOLD signal intensity for each calf muscle VOI. Image intensity values were expressed as a percent change from resting baseline signal intensity (i.e., image intensity value – baseline SI ÷ baseline SI). Four parameters were evaluated during transient ischemia and reactive hyperemia. Parameters included: (1) minimum ischemic value (MIV); (2) peak hyperemic value (PHV), defined as the highest 3‐second average value recorded following cuff deflation; (3) time to peak (TTP), which refers to the time from cuff deflation to peak BOLD signal; and (4) time to recovery (TTR), which was the time required to return to baseline BOLD signal intensity. Test–retest repeatability was evaluated for each parameter within all calf muscle VOIs in sedentary control subjects.

### Statistical analyses

Analysis of variance (ANOVA) was used to identify differences in BOLD signal intensity responses between each calf muscle VOI (four factors: gastrocnemius, soleus, anterior tibialis, and peroneus longus) as well as between‐subject groups (three factors: controls, linemen, and receivers/backs). Normality was assessed using the D'Agostino and Pearson omnibus normality test. Paired analysis was performed for evaluating differences in BOLD parameters between study visits in the control group. Pearson's correlation coefficient was used to assess the relation between maximal jumping ability (maximal broad and vertical jump) and peak hyperemic value. Two control subjects were excluded from correlation analysis due to missing data related to jumping ability, and three athletes were excluded from analysis as outliers. Normality was assessed using the D'Agostino and Pearson omnibus normality test. All statistical analyses were performed using commercially available software (GraphPad Prism v6.0 for Mac OS X, GraphPad Software, La Jolla, CA). Statistical significance for all analyses was set at *P* < 0.05. All values are expressed as means ± SD unless stated otherwise.

## Results

### Subject demographics

Subjects were separated into three groups: linemen, defensive backs/wide receivers, and sedentary controls. Subject group characteristics are summarized in Table 1. Linemen presented with significantly greater body weight, height, body fat composition, and systolic blood pressure when compared to backs/receivers and control subjects. Control subjects presented with significantly higher resting heart rate values than both linemen and backs/receivers. Control subjects also possessed lower diastolic blood pressure when compared to both athlete subject groups. In the assessment of maximal jumping ability, backs/receivers demonstrated significantly greater vertical and broad jumping ability when compared to both linemen and control subjects, and linemen possessed significantly greater broad jumping ability compared to control subjects.

**Table 1:** Subject characteristics

	Linemen (*n* = 12)	Backs/Receivers (*n* = 11)	Controls (*n* = 10)
Age, years	20.9 ± 1.0	20.6 ± 1.2	21.8 ± 2.2
Body Weight, kg	119.2 ± 6.6^b^	86.9 ± 7.2	80.2 ± 16.2^a^
Height, cm	191.5 ± 4.8^b^	180.1 ± 11.2	179.7 ± 5.3^a^
Body composition, % body fat	23.5 ± 3.1^b^	10.7 ± 2.4	N/A
Resting HR, bpm	63.2 ± 7.5	57.8 ± 8.1	76.3 ± 8.9^a^^,^ b
Systolic BP, mmHg	138.8 ± 12.0^b^	128.0 ± 9.5	119.7 ± 12.8^a^
Diastolic BP, mmHg	82.2 ± 8.3	78.7 ± 6.7	69.9 ± 11.0^a^^,^ b
Maximum vertical jump, cm	56.6 ± 6.5^b^	72.8 ± 5.1	50.7 ± 8.1^b^
Maximum broad jump, cm	239.3 ± 12.8^b^	273.2 ± 12.3	201.6 ± 32.3^a^^,^ b

N/A, not available.

All values are means ± SD.

a Significantly different from linemen.

b Significantly different from backs/receivers (*P* < 0.05).

The IPAQ revealed no differences in the average weekly amount of moderate and vigorous intensity exercise between athlete subject groups, as well as similar weekly totals for hours spent walking and sitting between athlete groups (Table 2). Control subjects significantly differed from both athlete groups with regard to the amount of time performing moderate and vigorous intensity exercise, as well as the average number of hours spent per week sitting.

**Table 2:** International physical activity questionnaire responses

	Linemen (*n* = 12)	Backs/Receivers (*n* = 11)	Controls (*n* = 10)
Vigorous exercise, h/week	27.7 ± 8.4	28.0 ± 8.3	0.3 ± 0.7^a^^,^ b
Moderate exercise, h/week	6.5 ± 5.8	9.0 ± 12.0	0.5 ± 1.3^a^^,^ b
Walking, h/week	10.3 ± 7.6	9.4 ± 8.2	8.8 ± 10.1
Sitting, h/week	25.7 ± 13.5	27.6 ± 11.9	70.4 ± 28.8^a^^,^ b

All values are means ± SD.

a Significantly different from linemen.

b Significantly different from backs/receivers (*P* < 0.05).

### Test–retest repeatability

Analysis of quantitative BOLD indices between study visits in sedentary control subjects demonstrated repeatability of PHV, TTP, MIV, and TTR measures for each muscle group of interest (Table 3).

**Table 3:** Test–Retest repeatability in sedentary control subjects

	Visit 1	CV (%)	Visit 2	CV (%)	*P*‐value
Peak hyperemic value (%)					
Gastrocnemius	7.4 ± 3.5	47.1	6.2 ±3.0	48.2	0.4
Soleus	12.9 ± 5.8	45.0	10.4 ±4.6	44.0	0.4
Anterior Tibialis	7.7 ± 4.0	52.1	8.0 ±3.9	48.6	0.9
Peroneus Longus	9.2 ± 5.1	55.2	6.7 ±1.8	26.2	0.2
Minimum ischemic value (%)					
Gastrocnemius	−13.3 ± 4.3	32.7	−10.2 ± 3.1	30.1	0.1
Soleus	−12.4 ± 6.4	51.7	−10.4 ± 3.7	36.1	0.4
Anterior Tibiatis	−7.5 ± 3.4	45.5	−6.1 ± 2.1	34.9	0.4
Peroneus Longus	−9.4 ± 5.5	58.8	−6.3 ± 1.8	29.5	0.2
Time‐to‐Peak (sec)					
Gastrocnemius	28.5 ± 5.6	19.5	29.3 ± 15.9	20.3	0.7
Soleus	21.0 ± 2.8	13.2	21.4 ± 7.9	37.1	0.9
Anterior Tibialis	26.3 ± 5.0	19.1	28.6 ± 9.5	33.2	0.4
Peroneus Longus	25.1 ± 6.6	26.3	29.3 ± 8.3	28.4	0.3
Time‐to‐Recovervy (sec)					
Gastrocnemius	129.4 ± 28.5	22.1	134.6± 37.8	28.1	0.6
Soleus	134.1 ± 21.5	16.0	139.5 ± 55.6	39.8	0.7
Anterior Tibialis	136.5 ± 40.2	29.4	153.4 ± 52.7	34.3	0.5
Peroneus Lougus	140.6 ± 48.4	34.4	124.9 ± 30.2	24.2	0.5

CV, coefficient of variation

All values are means ± SD. *N* = 10 subjects.

### BOLD analysis between‐subject groups

**Figure 1:**
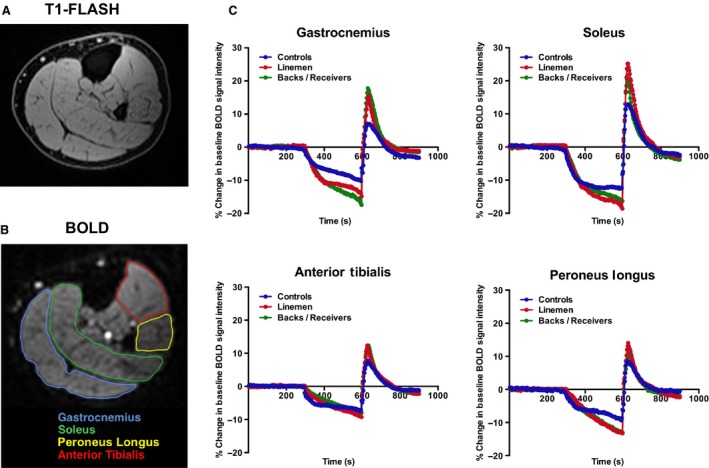
Regional assessment of dynamic changes in skeletal muscle tissue oxygenation during cuff occlusion and reactive hyperemia. (A) Anatomical T1‐FLASH images were used to identify and guide segmentation of lower extremity calf muscles. (B) Muscle groups of interest were segmented on T2*‐weighted BOLD images to generate (C) averaged dynamic time‐course data in each muscle group for each of the three subject groups. Arrows indicate time of cuff inflation (initialization of ischemia) and deflation (start of reactive hyperemia phase). Plotted data represents average values for all subjects within each group.

**Figure 2:**
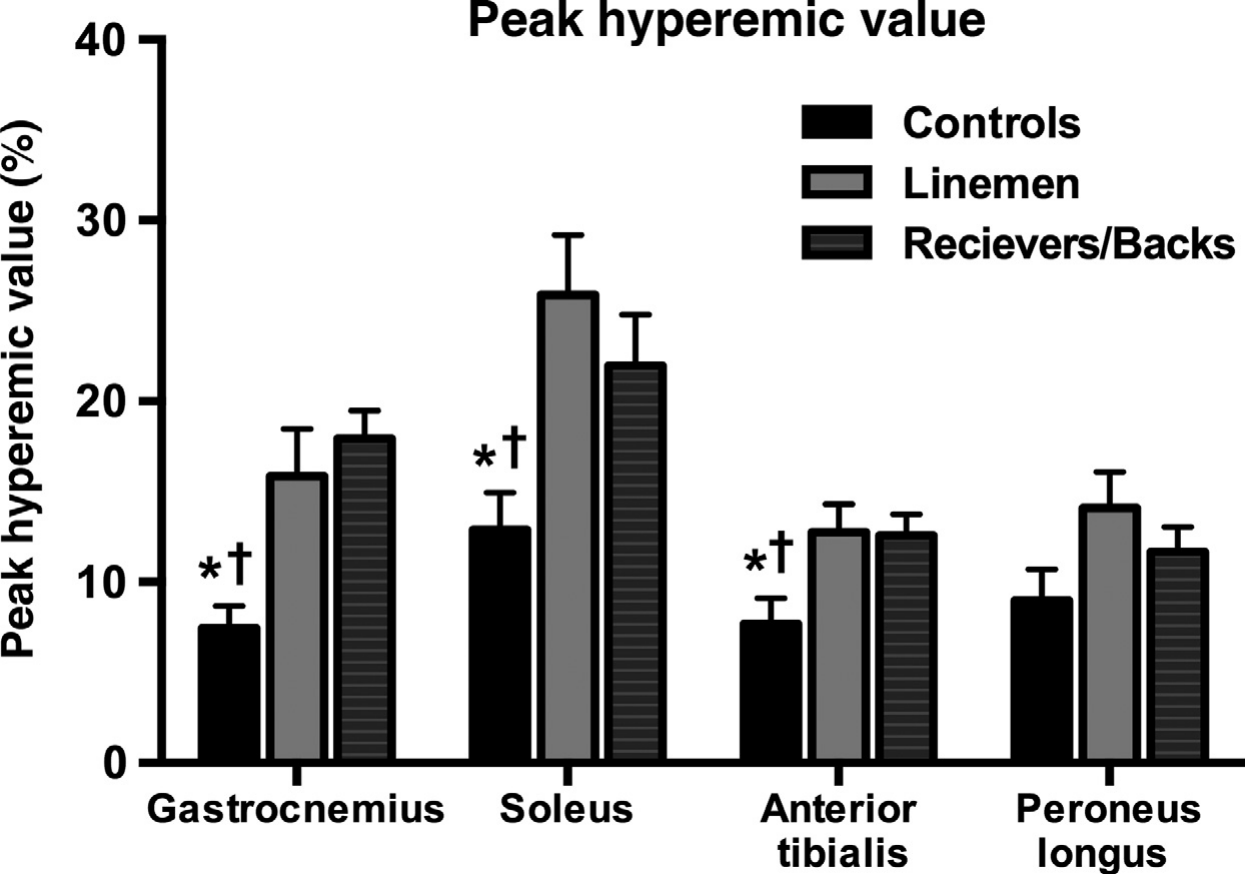
Evaluation of peak hyperemic value between‐subject groups. All values are means ± SD. *significantly different from linemen. ^†^significantly different from backs/receivers (*P* < 0.05).

Analysis of dynamic changes in BOLD signal between‐subject groups within specific muscle groups (gastrocnemius, soleus, anterior tibialis, and peroneus longus) revealed regional variability in the time‐course responses to cuff occlusion and reactive hyperemia (Fig. 1). Specifically, significantly higher PHVs were identified in both athlete groups for the gastrocnemius, soleus, and anterior tibialis muscle groups when compared to control subjects. No significant differences were found between any subject groups in evaluating the PHV of the peroneus longus (Fig. 2). Additionally, no significant differences were found between‐subject groups for all other evaluated BOLD indices (i.e., MIV, TTP, TTR; data not shown).

### BOLD analysis between skeletal muscle groups

**Figure 3:**
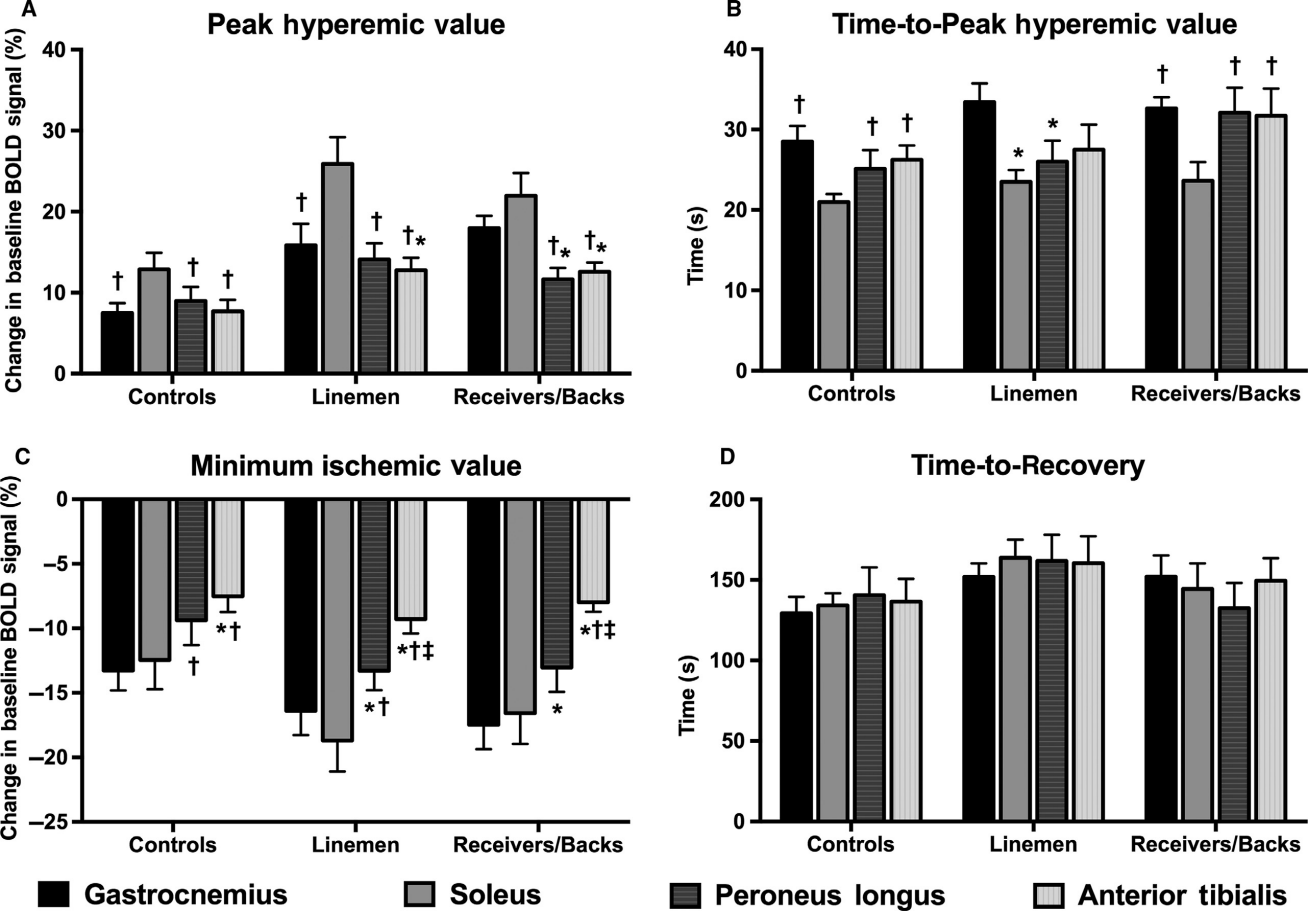
Regional evaluation and comparison of quantitative BOLD indices between calf muscles of interest. All values are means ± SD. *significantly different from gastrocnemius. ^†^significantly different from soleus. ^‡^significantly different from peroneus longus (*P* < 0.05).

All quantitative BOLD indices were assessed and compared across each muscle group of interest (Fig. 3). In the assessment of PHV in control subjects, the soleus muscle demonstrated a significantly higher PHV than all other muscle groups (Fig. 3A). The soleus muscle also demonstrated a significantly higher PHV than all other muscle groups in the linemen. Additionally, the gastrocnemius of linemen exhibited a higher PHV than the anterior tibialis muscle group. In the receivers/backs subject group, both the gastrocnemius and soleus muscles exhibited higher PHVs than the peroneus longus and anterior tibialis muscles.

In the assessment of TTP for the hyperemic value of control subjects and receivers/backs, the soleus muscle demonstrated a significantly faster TTP when compared to all other muscle groups (Fig. 3B). In the linemen subject group, the soleus and peroneus longus muscles both exhibited a significantly faster TTP than the gastrocnemius muscle group.

Evaluation of MIV demonstrated a similar trend between muscle groups for all three subject groups, where the gastrocnemius and soleus muscles on average exhibited the lowest MIVs during the cuff occlusion phase of our BOLD imaging protocol when compared to the peroneus longus and anterior tibialis muscles (Fig. 3C).

In the evaluation of TTR following reactive hyperemia, no significant differences were found between any muscle groups within any of the subject groups (Fig. 3D).

### Relationship between BOLD response and jumping ability

**Figure 4:**
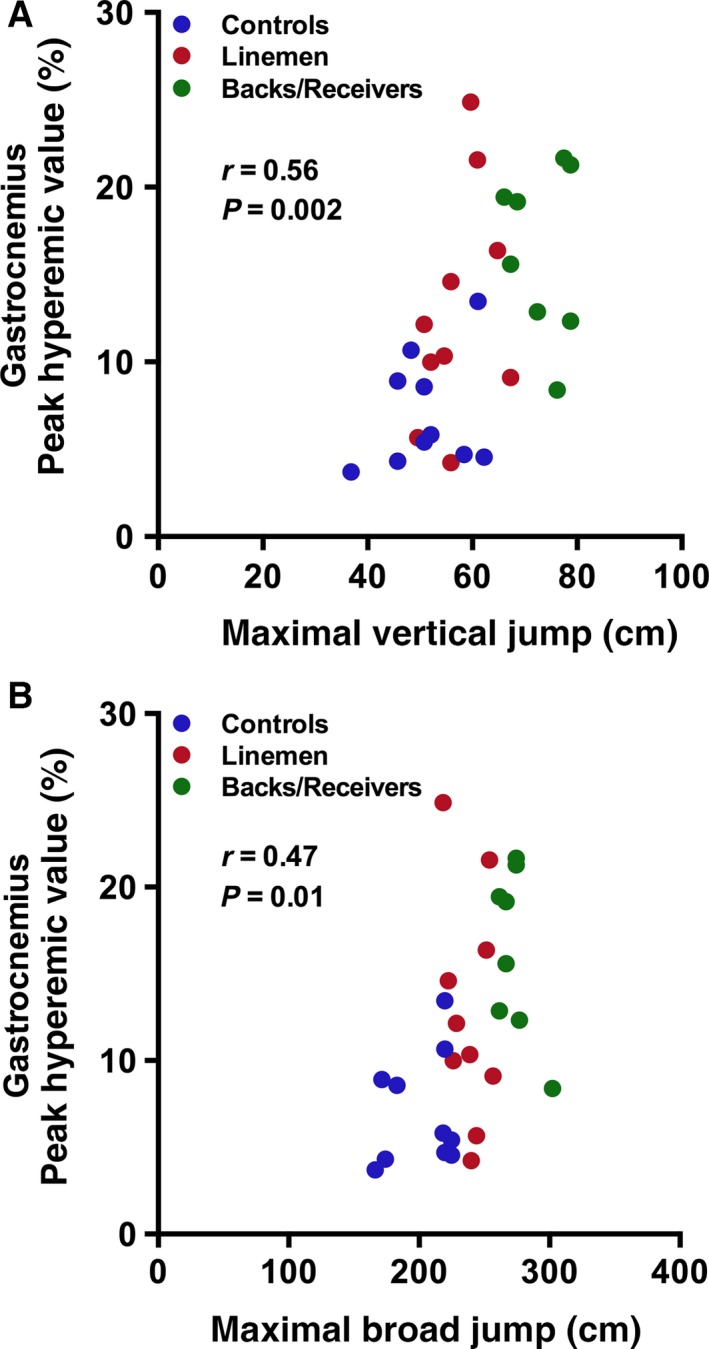
Relationship between peak hyperemic value of the gastrocnemius muscle and maximal vertical and broad jump. Peak hyperemic BOLD response in the gastrocnemius was significantly and positively related to both (A) vertical and (B) broad jumping ability. *N* = 28 subjects.

The PHV of the gastrocnemius muscle was significantly and positively related to both maximal vertical (*r *= 0.56; *P* = 0.002) and broad jump (*r *= 0.47; *P* = 0.01; Fig. 4). No other significant relationships were found between muscle‐specific BOLD responses and maximal jumping ability.

## Discussion

In this study, we have demonstrated for the first time the utility of BOLD MR imaging for evaluating differences in peak tissue oxygenation of lower extremity skeletal muscles between athletes and sedentary healthy control subjects by using a cuff occlusion and reactive hyperemia paradigm (Fig. 2). In addition to noninvasively identifying significant differences in regional microvascular tissue oxygenation between‐subject groups of varying physical activity levels, we have demonstrated significantly different vascular responses between muscle groups within the same‐subject groups (Fig. 3). Furthermore, we have identified a significant relationship between peak reactive hyperemia of the gastrocnemius muscle and maximal jumping ability, indicating that BOLD MR imaging may provide a noninvasive tool for tracking serial improvements in lower extremity function.

Previous studies have found higher BOLD responses in physically active versus sedentary control subjects following 1‐sec maximal voluntary contractions (Towse et al. 2005), and that peak BOLD responses after maximal muscle contraction are also significantly correlated with physical activity level (active vs. inactive) (Towse et al. 2011). However, prior studies evaluating the effects of physical activity on the BOLD signal have only assessed the anterior tibialis muscle in response to a single maximal muscle contraction paradigm. Given that research subjects could display differences in muscle contraction patterns, as well as the possibility for preexisting differences in vascular density between lower extremity muscle groups, we sought to evaluate a reactive hyperemia paradigm that would be capable of generating a more diffuse hyperemic BOLD response across all muscles of the calf in an effort to assess muscle group‐specific differences in tissue oxygenation between athletes and sedentary subjects. Using our reactive hyperemia approach to assess regional BOLD responses, we found that both athlete groups (linemen and backs/receivers) possessed higher PHVs than sedentary control subjects in the gastrocnemius, soleus, and anterior tibialis muscle groups, with no significant differences in PHV between athletes and sedentary controls for the peroneus longus muscle (Fig. 2). This finding suggests that specific lower extremity muscles may be more susceptible to exercise‐induced changes in tissue oxygenation and increased vascular density. These significant differences in peak BOLD responses between muscle groups also stresses the value of assessing multiple muscle groups in future investigations evaluating skeletal muscle adaptation to exercise training. In addition to comparing BOLD responses between athletes and sedentary controls, we found no significant differences between linemen and backs/receivers for any of the BOLD indices of vascular function. This finding is not too surprising given the similarities in physical activity levels of our athlete groups, as well as prior studies suggesting that exercise training‐induced changes in vascular function may be less apparent in younger athletes (Montero et al. 2014).

In the evaluation of muscle‐specific differences in our quantitative BOLD indices, the soleus muscle was found to have a higher PHV than other muscle groups, a finding that persisted in both athletes and sedentary control subjects (Fig. 3A). This finding is in agreement with prior studies (Ledermann et al. 2006a; Schulte et al. 2008) using similar reactive hyperemia paradigms and may be indicative of inherently high levels of vascular density in the soleus muscle group. In support of this argument, the soleus muscle also revealed a trend toward possessing a faster TTP than other muscle groups (Fig. 3B), which may be due to greater intravascular filling space to accommodate hyperemic blood flow, and thus suggestive of higher vascular density. In addition to demonstrating differences in PHV and TTP, the soleus, along with the gastrocnemius muscle, exhibited the lowest MIVs during the cuff occlusion phase of the imaging protocol (Fig. 3C). This finding indicates that the soleus and gastrocnemius muscles may be most susceptible to dramatic changes in tissue ischemia in the setting of acute arterial occlusion. However, in spite of the regional heterogeneity displayed across muscle groups during cuff occlusion and reactive hyperemia, no significant differences in TTR were found between muscles (Fig. 3D), suggesting that no differences in functional recovery exist between muscle groups. Future studies evaluating dynamic changes in BOLD signal during cuff occlusion and reactive hyperemia may be elucidated by incorporation of time‐of‐flight imaging or contrast‐enhanced MR angiography to provide complementary information related to tissue vascularization, and thus offer an explanation for the regional variations and adaptation in skeletal muscle oxygenation within athletes exposed to chronic exercise.

In evaluating the relationship between maximal vertical and broad jumping ability and peak BOLD response during reactive hyperemia (i.e., PHV), we found a significant and positive relationship between PHV of the gastrocnemius muscle and jumping ability, which existed for both vertical (Fig. 4A) and broad jump (Fig. 4B). However, the PHV of other muscle groups did not demonstrate a similar relationship with regard to jumping ability. This finding may be related to the important role that the gastrocnemius muscle is thought to play in vertical jumping exercise (van Soest et al. 1993), and may also be associated with exercise‐induced changes in fiber‐type composition of the gastrocnemius. Future application of BOLD MR imaging in patients undergoing exercise therapy for improved lower extremity function could provide opportunities for better evaluating this relationship and elucidate the functional relationship between peak vascular responses of individual muscles and functional capacity.

Collectively, the results of this study suggest that BOLD MR imaging is capable of detecting regional differences in microvascular skeletal muscle tissue oxygenation between athletes and sedentary subjects. Additionally, the peak BOLD response to reactive hyperemia may be closely related to functional capacity of lower extremity skeletal muscle. Future application of BOLD MR imaging may be useful for noninvasive evaluation of the serial response to exercise training or therapy in a variety of patient populations.

## Conflicts of Interest

The authors have no conflicts of interest to disclose.
